# Butyl­trichlorido{2-[(diisopropyl­ammonio)­meth­yl]phen­yl}tin(IV) dichloro­methane monosolvate

**DOI:** 10.1107/S1600536810050713

**Published:** 2010-12-11

**Authors:** Adina Rotar, Richard A. Varga, Malgorzata Staninska

**Affiliations:** a Faculty of Chemistry and Chemical Engineering, Babes-Bolyai University, 11 Arany Janos St, RO-400028, Cluj Napoca, Romania

## Abstract

The title compound, [Sn(C_4_H_9_)(C_13_H_21_N)Cl_3_]·CH_2_Cl_2_, was obtained by recrystallization of [2-(diisopropyl­amino­meth­yl)phen­yl]tin(IV) butyl dichloride from a CH_2_Cl_2_/*n*-hexane mixture (1:4 *v*/*v*) in the presence of ambient moisture. Partial hydrolysis led to the title compound, the hydro­chloric acid adduct of the dichloride, having a penta­coordinated Sn atom with a trigonal–bipyramidal C_2_SnCl_3_ core. The N atom of the 2-[(diisopropyl­ammonio)­meth­yl]phenyl ligand forms a strong intra­molecular N—H⋯Cl hydrogen bond, resulting in a zwitterionic species, [2-(^i^Pr_2_HN^+^CH_2_)C_6_H_4_]SnBuCl_3_^−^·CH_2_Cl_2_. Disorder was found in the *n*-butyl group, which was refined as disordered over three positions, with site occupancies of 0.22 (1), 0.51 (1) and 0.27 (2).

## Related literature

For related tin(IV) compounds, see: Varga *et al.* (2001 ▶, 2005 ▶, 2006 ▶); Varga & Silvestru (2007 ▶); Rotar *et al.* (2007 ▶, 2009 ▶); Rotar, Schuermann *et al.* (2008 ▶); Rotar, Varga & Silvestru (2008 ▶); Švec *et al.* (2010 ▶).
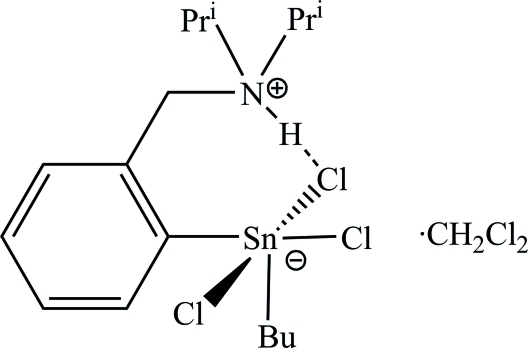


## Experimental

### 

#### Crystal data

[Sn(C_4_H_9_)(C_13_H_21_N)Cl_3_]·CH_2_Cl_2_
*M* *_r_* = 558.39
Triclinic, 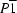

*a* = 10.654 (8) Å
*b* = 11.093 (9) Å
*c* = 12.406 (10) Å
α = 115.594 (13)°
β = 100.767 (15)°
γ = 97.176 (15)°
*V* = 1263.5 (17) Å^3^
*Z* = 2
Mo *K*α radiation
μ = 1.54 mm^−1^
*T* = 297 K
0.45 × 0.20 × 0.18 mm

#### Data collection

Bruker SMART APEX CCD area-detector diffractometer
Absorption correction: multi-scan (Bruker, 2000 ▶) *T*_min_ = 0.544, *T*_max_ = 0.769
9126 measured reflections
4416 independent reflections
3122 reflections with *I* > 2σ(*I*)
*R*_int_ = 0.074

#### Refinement

*R*[*F* ^2^ > 2σ(*F* ^2^)] = 0.076
*wR*(*F* ^2^) = 0.213
*S* = 1.02
4416 reflections
257 parameters
40 restraints
H atoms treated by a mixture of independent and constrained refinement
Δρ_max_ = 1.43 e Å^−3^
Δρ_min_ = −0.73 e Å^−3^

### 

Data collection: *SMART* (Bruker, 2000 ▶); cell refinement: *SAINT-Plus* (Bruker, 2001 ▶); data reduction: *SAINT-Plus*; program(s) used to solve structure: *SHELXS97* (Sheldrick, 2008 ▶); program(s) used to refine structure: *SHELXL97* (Sheldrick, 2008 ▶); molecular graphics: *DIAMOND* (Brandenburg & Putz, 2006 ▶); software used to prepare material for publication: *publCIF* (Westrip, 2010 ▶).

## Supplementary Material

Crystal structure: contains datablocks I, global. DOI: 10.1107/S1600536810050713/zl2332sup1.cif

Structure factors: contains datablocks I. DOI: 10.1107/S1600536810050713/zl2332Isup2.hkl

Additional supplementary materials:  crystallographic information; 3D view; checkCIF report

## Figures and Tables

**Table 1:** Hydrogen-bond geometry (Å, °)

*D*—H⋯*A*	*D*—H	H⋯*A*	*D*⋯*A*	*D*—H⋯*A*
N1—H1⋯Cl2	0.86 (1)	2.37 (7)	3.208 (8)	165 (8)

**Fig. 1.**
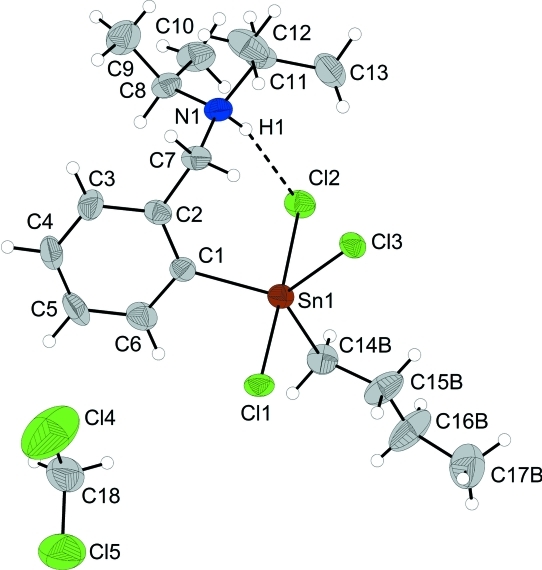
View of the title compound showing the atom-numbering scheme and the intramolecular hydrogen bond (black dashed line) with only the major component for the disordered n-butyl group. Displacement ellipsoids are drawn at the 30% probability level and H atoms as spheres of arbitrary radii.

**Fig. 2.**
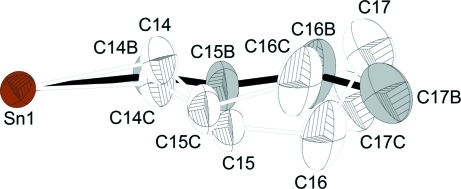
View of the disordered n-butyl group showing the atom-numbering scheme (major component with grey and the two minor components with white). Displacement ellipsoids are drawn at the 30% probability level (H atoms were omitted for clarity).

## supplementary crystallographic information

### Comment

During our work on hypercoordinated organotin(IV) compounds with [2-(*R*_2_NCH_2_)C_6_H_4_]Sn fragments (Varga *et al.*, 2001, 2005, 2006, Rotar *et al.*, 2007, 2008, 2009) the title compound was isolated.

In an attempt to obtain single-crystals of the dichloride [2-(^i^Pr_2_NCH_2_)C_6_H_4_]SnBuCl_2_ (1), recrystallization from a CH_2_Cl_2_/n-hexane mixture (1:4) in the presence of air afforded the HCl adduct 1.HCl.CH_2_Cl_2_. The nitrogen atom of the *L*^CN^ ligand [*L*^CN^ = 2-(diisopropylaminomethyl)phenyl] is protonated and a new Sn—Cl bond is simultaneously formed, thus leading to the formation of a zwitterionic species, [2-(^i^Pr_2_HN^+^CH_2_)C_6_H_4_]SnBuCl_3_^–^.CH_2_Cl_2_. The presence of the HCl is probably due to partial hydrolysis of the diorganotin(IV) dihalide in the presence of ambient moisture from air.

The central tin atom is pentacoordinated with a distorted trigonal bipyramidal geometry (Fig. 1). The axial positions are occupied by two chlorine atoms [Cl1—Sn1—Cl2 = 178.50 (7)°], while the two carbon atoms from the two organic groups and a chlorine atom are placed in equatorial positions. The angles in the equatorial C_2_SnCl system are situated in the range between 97.4 (13) and 145.8 (14) °, showing a strong deviation from the ideal value of 120°. The Cl2 atom forms a strong intramolecular hydrogen bond with H1 from the nitrogen atom [Cl2···H1 = 2.37 (7) Å].

Disorder was found in the *n*-butyl group. This was resolved over three positions with the components of the disorder having site a occupancy ratio of 0.21 (1):0.51 (2):0.27 (2) (see Refinement section; Fig. 2).

### Experimental

A solution of BuLi in hexane (10.5 ml, 1.6*M*, 20% excess) was added dropwise to a stirred solution of 2-(*N*,*N*-diisopropylaminomethyl)benzene bromide (3.7 g, 13.7 mmol) in 100 ml of anhydrous hexane at room temperature under argon using Schlenk techniques. The reaction mixture was refluxed under stirring for four hours and then allowed to reach room temperature. The obtained liquid product was added dropwise under stirring to a cooled (195 K, -78°C) solution of BuSnCl_3_ (1.8 ml, 11 mmol) in 50 ml of anhydrous hexane. After the organolithiun compound was added, the reaction mixture was stirred for 1 h at 195 K (-78°C), then overnight to reach room temperature. The solvent was removed *in vacuo.* The oily residue was recrystallized from CH_2_Cl_2_/n-hexane, in presence of air moisture, resulting in the isolation of [2-(^i^Pr_2_HN^+^CH_2_)C_6_H_4_]SnBuCl_3_^–^. CH_2_Cl_2_ (1.67 g, 32.9%).

^1^H NMR (300 MHz, CDCl_3_, 293.5K): 0.98 t (3*H*, SnCH_2_CH_2_CH_2_C*H*_3_, ^3^*J*_HH_ = 7.3 Hz), 1.28 d (6H_A_, NCH(C*H*_3_)_2_, ^3^*J*_HH_ = 6.7 Hz), 1.47 d (6H_B_, NCH(C*H*_3_)_2_, ^3^*J*_HH_ = 6.8 Hz), 1.52 s (2*H*, SnCH_2_CH_2_C*H*_2_CH_3_, ^3^*J*_HH_ = 7.4 Hz), 2.01 quin (2*H*, SnCH_2_C*H*_2_CH_2_CH_3_, ^3^*J*_HH_ = 8.2 Hz), 2.23 t (2*H*, SnC*H*_2_CH_2_CH_2_CH_3_, ^3^*J*_HH_ = 8.1, ^2^*J*_SnH_ = 81.4 Hz), 3.50 H (2*H*, NC*H*(CH_3_)_2_, ^3^*J*_HH_ = 6.5 Hz), 3.96 d (2*H*, –C*H*_2_–, ^3^*J*_HH_ = 4.3 Hz), 7.49*m* (2*H*, *H*_4,5_, C_6_*H*_4_), 7.65*m* (1*H*, *H*_3_, C_6_*H*_4_), 7.71*m* (1*H*, *H*_6_, C_6_*H*_4_), 9.22 s,br (1*H*, N*H*).

^13^C NMR (75.4 MHz, CDCl_3_, 293.5K): 13.68 s (SnCH_2_CH_2_CH_2_*C*H_3_, ^4^*J*_SnC_ = 7.8 Hz), 18.73 s (NCH(*C*H_3_)_2_ [B]), 19.11 s (NCH(*C*H_3_)_2_ [A]), 25.95 s (SnCH_2_CH_2_*C*H_2_CH_3_, ^3^*J*_SnC_ = 130.6 Hz), 27.47 s (SnCH_2_*C*H_2_CH_2_CH_3_, ^2^*J*_SnC_ = 51.1 Hz), 37.40 s,br (Sn*C*H_2_CH_2_CH_2_CH_3_), 51.07 s (-*C*H_2_–), 52.90 s (N*C*H(CH_3_)_2_), 129.71 s (C_5_, C_6_*H*_4_, ^3^*J*_SnC_ 51.1 = Hz), 129.86 s (C_4_, C_6_*H*_4_, ^4^*J*_SnC_ = 16.3 Hz), 132.15 s (C_1_, C_6_*H*_4_), 134.77 s (C_3_, C_6_*H*_4_, ^3^*J*_SnC_ = 61.8 Hz), 136.11 s (C_6_, C_6_*H*_4_, ^2^*J*_SnC_ = 80.1 Hz), 154.98 s (C_2_, C_6_*H*_4_).

### Refinement

All hydrogen atoms were placed in calculated positions using a riding model, with C—H = 0.93–0.98 Å and with *U*_iso_= 1.2 or 1.5*U*_eq_ (C) for H. The methyl groups were allowed to rotate but not to tip. The H1 atom bonded to N1 was found in a difference map and refined with a restrained N—H distance of 0.86 (1) Å.

The *n*-butyl group was found to be severly disordered. Attempts to refine the chain as disordered over two moieties (with appropriate distance restraints for the C—C bonds) did not give satisfactory results with ADPs of neighboring atoms being icompatible even after application of severe restraints for the thermal ellipsoids. Disorder over three moieties allowed to avoid these problems. In the final refinement the butyl chain was refined as disordered over three sites with equivalent bonds from the disordered components restrained to have similar lengths length. The same *U*_ij_ parameters were used for atom C14/C14B/C14C, C15/C15B/C15C, C16/C16B/C16C and C17/C17B/C17C, leading to refined site occupancies of 0.21 (1):0.51 (2):0.27 (2).

### Crystal data

**Table:**

[Sn(C_4_H_9_)(C_13_H_21_N)Cl_3_]·CH_2_Cl_2_	*Z* = 2
*M**_r_* = 558.39	*F*(000) = 564
Triclinic, *P*1	*D*_x_ = 1.468 Mg m^−^^3^
Hall symbol: -p 1	Mo *K*α radiation, λ = 0.71073 Å
*a* = 10.654 (8) Å	Cell parameters from 1665 reflections
*b* = 11.093 (9) Å	θ = 2.4–20.1°
*c* = 12.406 (10) Å	µ = 1.54 mm^−^^1^
α = 115.594 (13)°	*T* = 297 K
β = 100.767 (15)°	Block, colourless
γ = 97.176 (15)°	0.45 × 0.20 × 0.18 mm
*V* = 1263.5 (17) Å^3^	

### Data collection

**Table:**

Bruker SMART APEX CCD area-detector diffractometer	4416 independent reflections
Radiation source: fine-focus sealed tube	3122 reflections with *I* > 2σ(*I*)
graphite	*R*_int_ = 0.074
phi and ω scans	θ_max_ = 25.0°, θ_min_ = 2.0°
Absorption correction: multi-scan (Bruker, 2000)	*h* = −12→12
*T*_min_ = 0.544, *T*_max_ = 0.769	*k* = −13→13
9126 measured reflections	*l* = −14→14

### Refinement

**Table:**

Refinement on *F*^2^	Primary atom site location: structure-invariant direct methods
Least-squares matrix: full	Secondary atom site location: difference Fourier map
*R*[*F*^2^ > 2σ(*F*^2^)] = 0.076	Hydrogen site location: inferred from neighbouring sites
*wR*(*F*^2^) = 0.213	H atoms treated by a mixture of independent and constrained refinement
*S* = 1.02	*w* = 1/[σ^2^(*F*_o_^2^) + (0.1038*P*)^2^ + 0.0754*P*] where *P* = (*F*_o_^2^ + 2*F*_c_^2^)/3
4416 reflections	(Δ/σ)_max_ = 0.008
257 parameters	Δρ_max_ = 1.43 e Å^−^^3^
40 restraints	Δρ_min_ = −0.73 e Å^−^^3^

### Special details

**Table:**

Geometry. All e.s.d.'s (except the e.s.d. in the dihedral angle between two l.s. planes) are estimated using the full covariance matrix. The cell e.s.d.'s are taken into account individually in the estimation of e.s.d.'s in distances, angles and torsion angles; correlations between e.s.d.'s in cell parameters are only used when they are defined by crystal symmetry. An approximate (isotropic) treatment of cell e.s.d.'s is used for estimating e.s.d.'s involving l.s. planes.
Refinement. Refinement of *F*^2^ against ALL reflections. The weighted *R*-factor *wR* and goodness of fit *S* are based on *F*^2^, conventional *R*-factors *R* are based on *F*, with *F* set to zero for negative *F*^2^. The threshold expression of *F*^2^ > σ(*F*^2^) is used only for calculating *R*-factors(gt) *etc*. and is not relevant to the choice of reflections for refinement. *R*-factors based on *F*^2^ are statistically about twice as large as those based on *F*, and *R*- factors based on ALL data will be even larger.

### Fractional atomic coordinates and isotropic or equivalent isotropic displacement parameters (Å^2^)

**Table:**

	*x*	*y*	*z*	*U*_iso_*/*U*_eq_	Occ. (<1)
C1	0.4960 (8)	0.7632 (8)	0.8171 (7)	0.0490 (19)	
C2	0.4078 (8)	0.8089 (8)	0.8876 (7)	0.052 (2)	
C3	0.4102 (10)	0.7820 (10)	0.9900 (7)	0.068 (3)	
H3	0.3545	0.8145	1.0401	0.082*	
C4	0.4950 (11)	0.7076 (11)	1.0147 (9)	0.079 (3)	
H4	0.4942	0.6882	1.0804	0.095*	
C5	0.5813 (11)	0.6613 (11)	0.9442 (10)	0.080 (3)	
H5	0.6392	0.6124	0.9626	0.096*	
C6	0.5798 (10)	0.6891 (10)	0.8458 (9)	0.070 (3)	
H6	0.6369	0.6570	0.7973	0.084*	
C7	0.3141 (8)	0.8942 (8)	0.8704 (7)	0.054 (2)	
H7A	0.3545	0.9547	0.8424	0.065*	
H7B	0.2999	0.9514	0.9501	0.065*	
C8	0.1153 (9)	0.6964 (9)	0.7985 (8)	0.061 (2)	
H8	0.1822	0.6500	0.8175	0.073*	
C9	0.0569 (11)	0.7551 (12)	0.9098 (10)	0.093 (3)	
H9A	0.1267	0.8024	0.9852	0.139*	
H9B	0.0012	0.6814	0.9118	0.139*	
H9C	0.0064	0.8181	0.9019	0.139*	
C10	0.0112 (9)	0.5909 (11)	0.6802 (9)	0.084 (3)	
H10A	−0.0587	0.6325	0.6632	0.126*	
H10B	−0.0232	0.5140	0.6910	0.126*	
H10C	0.0501	0.5602	0.6119	0.126*	
C11	0.0904 (9)	0.8991 (10)	0.7562 (8)	0.067 (2)	
H11	0.0007	0.8537	0.7457	0.080*	
C12	0.1211 (12)	1.0427 (11)	0.8664 (10)	0.098 (4)	
H12A	0.1148	1.0351	0.9395	0.147*	
H12B	0.0593	1.0926	0.8498	0.147*	
H12C	0.2084	1.0905	0.8791	0.147*	
C13	0.0929 (13)	0.9059 (15)	0.6384 (10)	0.109 (4)	
H13A	0.0260	0.9499	0.6199	0.163*	
H13B	0.0769	0.8145	0.5716	0.163*	
H13C	0.1773	0.9575	0.6483	0.163*	
C14	0.571 (6)	0.712 (5)	0.490 (3)	0.065 (6)	0.22
H14A	0.5530	0.6136	0.4589	0.078*	0.22
H14B	0.5079	0.7296	0.4342	0.078*	0.22
C15	0.702 (6)	0.754 (8)	0.477 (7)	0.089 (8)	0.22
H15A	0.7620	0.7795	0.5565	0.107*	0.22
H15B	0.7010	0.8400	0.4735	0.107*	0.22
C16	0.772 (6)	0.681 (6)	0.384 (5)	0.124 (10)	0.22
H16A	0.8528	0.7432	0.3967	0.148*	0.22
H16B	0.7955	0.6044	0.3948	0.148*	0.22
C17	0.686 (9)	0.632 (10)	0.258 (7)	0.127 (10)	0.22
H17A	0.6103	0.6701	0.2635	0.191*	0.22
H17B	0.6587	0.5331	0.2181	0.191*	0.22
H17C	0.7333	0.6591	0.2105	0.191*	0.22
C14B	0.587 (10)	0.664 (4)	0.511 (7)	0.065 (6)	0.51
H14C	0.6522	0.6240	0.5402	0.078*	0.51
H14D	0.5129	0.5900	0.4515	0.078*	0.51
C15B	0.644 (4)	0.733 (3)	0.446 (3)	0.089 (8)	0.51
H15C	0.7198	0.8063	0.5038	0.107*	0.51
H15D	0.5797	0.7743	0.4161	0.107*	0.51
C16B	0.684 (4)	0.634 (3)	0.340 (4)	0.124 (10)	0.51
H16C	0.7401	0.5852	0.3687	0.148*	0.51
H16D	0.6065	0.5662	0.2783	0.148*	0.51
C17B	0.755 (3)	0.699 (4)	0.280 (3)	0.127 (10)	0.51
H17D	0.6927	0.7125	0.2216	0.191*	0.51
H17E	0.8077	0.6408	0.2368	0.191*	0.51
H17F	0.8104	0.7862	0.3418	0.191*	0.51
C14C	0.600 (19)	0.687 (6)	0.512 (13)	0.065 (6)	0.27
H14E	0.6634	0.6401	0.5342	0.078*	0.27
H14F	0.5301	0.6190	0.4404	0.078*	0.27
C15C	0.663 (7)	0.794 (6)	0.486 (5)	0.089 (8)	0.27
H15E	0.7464	0.8457	0.5474	0.107*	0.27
H15F	0.6072	0.8569	0.4877	0.107*	0.27
C16C	0.685 (6)	0.724 (4)	0.360 (5)	0.124 (10)	0.27
H16E	0.6005	0.6957	0.3002	0.148*	0.27
H16F	0.7398	0.7924	0.3497	0.148*	0.27
C17C	0.744 (8)	0.603 (6)	0.324 (7)	0.127 (10)	0.27
H17G	0.6755	0.5202	0.2812	0.191*	0.27
H17H	0.7975	0.6054	0.3965	0.191*	0.27
H17I	0.7974	0.6042	0.2696	0.191*	0.27
C18	0.7688 (13)	0.2311 (16)	0.7029 (15)	0.131 (6)	
H18A	0.7283	0.1506	0.7073	0.157*	
H18B	0.7266	0.2221	0.6226	0.157*	
Cl1	0.75445 (19)	0.9363 (2)	0.79244 (19)	0.0594 (6)	
Cl2	0.2839 (2)	0.6520 (2)	0.5385 (2)	0.0644 (6)	
Cl3	0.4604 (2)	0.9945 (2)	0.65829 (19)	0.0549 (5)	
Cl4	0.7400 (7)	0.3745 (6)	0.8168 (7)	0.234 (4)	
Cl5	0.9241 (6)	0.2338 (7)	0.7127 (7)	0.214 (3)	
N1	0.1814 (7)	0.8103 (7)	0.7781 (6)	0.0557 (18)	
H1	0.197 (8)	0.773 (8)	0.707 (4)	0.067*	
Sn1	0.52253 (6)	0.79582 (6)	0.66440 (5)	0.0525 (3)	

### Atomic displacement parameters (Å^2^)

**Table:**

	*U*^11^	*U*^22^	*U*^33^	*U*^12^	*U*^13^	*U*^23^
C1	0.039 (5)	0.055 (5)	0.045 (4)	0.004 (4)	0.005 (4)	0.020 (4)
C2	0.043 (5)	0.055 (5)	0.047 (4)	0.010 (4)	0.004 (4)	0.017 (4)
C3	0.069 (6)	0.091 (7)	0.047 (5)	0.012 (5)	0.016 (4)	0.035 (5)
C4	0.096 (8)	0.100 (8)	0.074 (6)	0.043 (7)	0.026 (6)	0.064 (6)
C5	0.081 (8)	0.090 (7)	0.090 (7)	0.035 (6)	0.004 (6)	0.063 (6)
C6	0.072 (7)	0.070 (6)	0.076 (6)	0.035 (5)	0.025 (5)	0.034 (5)
C7	0.044 (5)	0.058 (5)	0.043 (4)	0.011 (4)	0.010 (4)	0.009 (4)
C8	0.047 (5)	0.066 (5)	0.067 (5)	0.005 (4)	0.019 (4)	0.029 (5)
C9	0.075 (8)	0.103 (8)	0.086 (7)	−0.008 (6)	0.015 (6)	0.042 (7)
C10	0.052 (6)	0.086 (7)	0.078 (7)	−0.007 (5)	0.005 (5)	0.017 (6)
C11	0.045 (5)	0.077 (6)	0.080 (6)	0.020 (5)	0.017 (5)	0.037 (5)
C12	0.094 (9)	0.075 (7)	0.100 (8)	0.049 (6)	0.010 (7)	0.017 (6)
C13	0.110 (10)	0.158 (12)	0.091 (8)	0.076 (9)	0.029 (7)	0.075 (9)
C14	0.087 (17)	0.046 (13)	0.066 (7)	0.035 (16)	0.034 (9)	0.019 (12)
C15	0.09 (2)	0.09 (2)	0.062 (19)	0.00 (2)	0.042 (19)	0.004 (15)
C16	0.15 (3)	0.081 (18)	0.16 (2)	0.004 (19)	0.10 (2)	0.06 (2)
C17	0.16 (3)	0.14 (3)	0.095 (16)	0.04 (2)	0.038 (18)	0.060 (18)
C14B	0.087 (17)	0.046 (13)	0.066 (7)	0.035 (16)	0.034 (9)	0.019 (12)
C15B	0.09 (2)	0.09 (2)	0.062 (19)	0.00 (2)	0.042 (19)	0.004 (15)
C16B	0.15 (3)	0.081 (18)	0.16 (2)	0.004 (19)	0.10 (2)	0.06 (2)
C17B	0.16 (3)	0.14 (3)	0.095 (16)	0.04 (2)	0.038 (18)	0.060 (18)
C14C	0.087 (17)	0.046 (13)	0.066 (7)	0.035 (16)	0.034 (9)	0.019 (12)
C15C	0.09 (2)	0.09 (2)	0.062 (19)	0.00 (2)	0.042 (19)	0.004 (15)
C16C	0.15 (3)	0.081 (18)	0.16 (2)	0.004 (19)	0.10 (2)	0.06 (2)
C17C	0.16 (3)	0.14 (3)	0.095 (16)	0.04 (2)	0.038 (18)	0.060 (18)
C18	0.094 (10)	0.156 (13)	0.208 (16)	0.038 (9)	0.044 (10)	0.139 (13)
Cl1	0.0326 (11)	0.0740 (13)	0.0603 (12)	0.0032 (10)	0.0069 (9)	0.0261 (11)
Cl2	0.0525 (14)	0.0652 (13)	0.0581 (12)	0.0074 (11)	0.0045 (10)	0.0194 (11)
Cl3	0.0529 (13)	0.0512 (11)	0.0716 (13)	0.0172 (10)	0.0157 (10)	0.0374 (10)
Cl4	0.316 (10)	0.177 (5)	0.300 (8)	0.125 (6)	0.220 (8)	0.120 (5)
Cl5	0.163 (5)	0.303 (7)	0.364 (8)	0.147 (5)	0.150 (5)	0.264 (7)
N1	0.045 (4)	0.066 (4)	0.052 (4)	0.016 (4)	0.016 (3)	0.022 (4)
Sn1	0.0492 (4)	0.0576 (4)	0.0541 (4)	0.0190 (3)	0.0164 (3)	0.0262 (3)

### Geometric parameters (Å, °)

**Table:**

C1—C6	1.385 (12)	C15—H15B	0.9700
C1—C2	1.396 (11)	C16—C17	1.48 (2)
C1—Sn1	2.137 (8)	C16—H16A	0.9700
C2—C3	1.421 (11)	C16—H16B	0.9700
C2—C7	1.506 (12)	C17—H17A	0.9600
C3—C4	1.376 (13)	C17—H17B	0.9600
C3—H3	0.9300	C17—H17C	0.9600
C4—C5	1.380 (14)	C14B—C15B	1.483 (18)
C4—H4	0.9300	C14B—Sn1	2.137 (12)
C5—C6	1.380 (13)	C14B—H14C	0.9700
C5—H5	0.9300	C14B—H14D	0.9700
C6—H6	0.9300	C15B—C16B	1.490 (18)
C7—N1	1.524 (11)	C15B—H15C	0.9700
C7—H7A	0.9700	C15B—H15D	0.9700
C7—H7B	0.9700	C16B—C17B	1.479 (18)
C8—N1	1.509 (11)	C16B—H16C	0.9700
C8—C10	1.529 (12)	C16B—H16D	0.9700
C8—C9	1.537 (14)	C17B—H17D	0.9600
C8—H8	0.9800	C17B—H17E	0.9600
C9—H9A	0.9600	C17B—H17F	0.9600
C9—H9B	0.9600	C14C—C15C	1.48 (2)
C9—H9C	0.9600	C14C—Sn1	2.139 (16)
C10—H10A	0.9600	C14C—H14E	0.9700
C10—H10B	0.9600	C14C—H14F	0.9700
C10—H10C	0.9600	C15C—C16C	1.49 (2)
C11—C13	1.500 (13)	C15C—H15E	0.9700
C11—C12	1.527 (13)	C15C—H15F	0.9700
C11—N1	1.535 (11)	C16C—C17C	1.48 (2)
C11—H11	0.9800	C16C—H16E	0.9700
C12—H12A	0.9600	C16C—H16F	0.9700
C12—H12B	0.9600	C17C—H17G	0.9600
C12—H12C	0.9600	C17C—H17H	0.9600
C13—H13A	0.9600	C17C—H17I	0.9600
C13—H13B	0.9600	C18—Cl5	1.632 (14)
C13—H13C	0.9600	C18—Cl4	1.721 (16)
C14—C15	1.481 (19)	C18—H18A	0.9700
C14—Sn1	2.141 (18)	C18—H18B	0.9700
C14—H14A	0.9700	Cl1—Sn1	2.547 (3)
C14—H14B	0.9700	Cl2—Sn1	2.608 (3)
C15—C16	1.485 (19)	Cl3—Sn1	2.406 (3)
C15—H15A	0.9700	N1—H1	0.857 (10)
			
C6—C1—C2	119.4 (8)	H17B—C17—H17C	109.5
C6—C1—Sn1	113.1 (6)	C15B—C14B—Sn1	114.6 (19)
C2—C1—Sn1	127.5 (6)	C15B—C14B—H14C	108.6
C1—C2—C3	118.7 (8)	Sn1—C14B—H14C	108.6
C1—C2—C7	125.5 (8)	C15B—C14B—H14D	108.6
C3—C2—C7	115.7 (8)	Sn1—C14B—H14D	108.6
C4—C3—C2	119.8 (9)	H14C—C14B—H14D	107.6
C4—C3—H3	120.1	C14B—C15B—C16B	111 (2)
C2—C3—H3	120.1	C14B—C15B—H15C	109.5
C3—C4—C5	121.5 (9)	C16B—C15B—H15C	109.5
C3—C4—H4	119.3	C14B—C15B—H15D	109.5
C5—C4—H4	119.3	C16B—C15B—H15D	109.5
C6—C5—C4	118.5 (9)	H15C—C15B—H15D	108.1
C6—C5—H5	120.7	C17B—C16B—C15B	113 (3)
C4—C5—H5	120.7	C17B—C16B—H16C	108.9
C5—C6—C1	122.1 (9)	C15B—C16B—H16C	108.9
C5—C6—H6	119.0	C17B—C16B—H16D	108.9
C1—C6—H6	119.0	C15B—C16B—H16D	108.9
C2—C7—N1	114.1 (7)	H16C—C16B—H16D	107.7
C2—C7—H7A	108.7	C16B—C17B—H17D	109.5
N1—C7—H7A	108.7	C16B—C17B—H17E	109.5
C2—C7—H7B	108.7	H17D—C17B—H17E	109.5
N1—C7—H7B	108.7	C16B—C17B—H17F	109.5
H7A—C7—H7B	107.6	H17D—C17B—H17F	109.5
N1—C8—C10	110.1 (7)	H17E—C17B—H17F	109.5
N1—C8—C9	110.6 (8)	C15C—C14C—Sn1	105 (3)
C10—C8—C9	111.8 (8)	C15C—C14C—H14E	110.8
N1—C8—H8	108.1	Sn1—C14C—H14E	110.8
C10—C8—H8	108.1	C15C—C14C—H14F	110.9
C9—C8—H8	108.1	Sn1—C14C—H14F	110.9
C8—C9—H9A	109.5	H14E—C14C—H14F	108.9
C8—C9—H9B	109.5	C14C—C15C—C16C	108 (4)
H9A—C9—H9B	109.5	C14C—C15C—H15E	110.2
C8—C9—H9C	109.5	C16C—C15C—H15E	110.2
H9A—C9—H9C	109.5	C14C—C15C—H15F	110.1
H9B—C9—H9C	109.5	C16C—C15C—H15F	110.1
C8—C10—H10A	109.5	H15E—C15C—H15F	108.5
C8—C10—H10B	109.5	C17C—C16C—C15C	121 (5)
H10A—C10—H10B	109.5	C17C—C16C—H16E	107.0
C8—C10—H10C	109.5	C15C—C16C—H16E	107.0
H10A—C10—H10C	109.5	C17C—C16C—H16F	107.0
H10B—C10—H10C	109.5	C15C—C16C—H16F	107.0
C13—C11—C12	111.4 (10)	H16E—C16C—H16F	106.8
C13—C11—N1	110.8 (8)	C16C—C17C—H17G	109.5
C12—C11—N1	112.6 (7)	C16C—C17C—H17H	109.5
C13—C11—H11	107.2	H17G—C17C—H17H	109.5
C12—C11—H11	107.2	C16C—C17C—H17I	109.5
N1—C11—H11	107.2	H17G—C17C—H17I	109.5
C11—C12—H12A	109.5	H17H—C17C—H17I	109.5
C11—C12—H12B	109.5	Cl5—C18—Cl4	114.1 (10)
H12A—C12—H12B	109.5	Cl5—C18—H18A	108.7
C11—C12—H12C	109.5	Cl4—C18—H18A	108.7
H12A—C12—H12C	109.5	Cl5—C18—H18B	108.7
H12B—C12—H12C	109.5	Cl4—C18—H18B	108.7
C11—C13—H13A	109.5	H18A—C18—H18B	107.6
C11—C13—H13B	109.5	C8—N1—C7	114.9 (7)
H13A—C13—H13B	109.5	C8—N1—C11	114.1 (7)
C11—C13—H13C	109.5	C7—N1—C11	113.3 (7)
H13A—C13—H13C	109.5	C8—N1—H1	108 (6)
H13B—C13—H13C	109.5	C7—N1—H1	105 (6)
C15—C14—Sn1	123 (4)	C11—N1—H1	100 (6)
C15—C14—H14A	106.6	C14B—Sn1—C1	126.9 (17)
Sn1—C14—H14A	106.6	C14B—Sn1—C14C	7(3)
C15—C14—H14B	106.6	C1—Sn1—C14C	132 (3)
Sn1—C14—H14B	106.6	C14B—Sn1—C14	19 (2)
H14A—C14—H14B	106.5	C1—Sn1—C14	145.8 (14)
C14—C15—C16	132 (5)	C14C—Sn1—C14	15 (5)
C14—C15—H15A	104.4	C14B—Sn1—Cl3	116.5 (18)
C16—C15—H15A	104.4	C1—Sn1—Cl3	116.5 (2)
C14—C15—H15B	104.4	C14C—Sn1—Cl3	111 (3)
C16—C15—H15B	104.4	C14—Sn1—Cl3	97.4 (13)
H15A—C15—H15B	105.6	C14B—Sn1—Cl1	93 (3)
C17—C16—C15	109 (7)	C1—Sn1—Cl1	89.0 (2)
C17—C16—H16A	109.9	C14C—Sn1—Cl1	89 (5)
C15—C16—H16A	109.9	C14—Sn1—Cl1	95.1 (16)
C15—C16—H16B	109.9	Cl3—Sn1—Cl1	90.96 (9)
H16A—C16—H16B	108.3	C14B—Sn1—Cl2	88 (3)
C16—C17—H17A	109.5	C1—Sn1—Cl2	89.5 (2)
C16—C17—H17B	109.5	C14C—Sn1—Cl2	92 (5)
H17A—C17—H17B	109.5	C14—Sn1—Cl2	86.3 (16)
C16—C17—H17C	109.5	Cl3—Sn1—Cl2	89.46 (9)
H17A—C17—H17C	109.5	Cl1—Sn1—Cl2	178.50 (7)
			
C6—C1—C2—C3	2.3 (12)	C15B—C14B—Sn1—C1	−158 (5)
Sn1—C1—C2—C3	−176.8 (6)	C15B—C14B—Sn1—C14	29 (6)
C6—C1—C2—C7	177.9 (8)	C15B—C14B—Sn1—Cl3	26 (8)
Sn1—C1—C2—C7	−1.2 (12)	C15B—C14B—Sn1—Cl1	−67 (7)
C1—C2—C3—C4	−2.4 (13)	C15B—C14B—Sn1—Cl2	114 (7)
C7—C2—C3—C4	−178.4 (9)	C6—C1—Sn1—C14B	30 (4)
C2—C3—C4—C5	1.8 (16)	C2—C1—Sn1—C14B	−151 (4)
C3—C4—C5—C6	−1.0 (17)	C6—C1—Sn1—C14C	24 (7)
C4—C5—C6—C1	0.9 (16)	C2—C1—Sn1—C14C	−157 (7)
C2—C1—C6—C5	−1.5 (14)	C6—C1—Sn1—C14	34 (3)
Sn1—C1—C6—C5	177.7 (8)	C2—C1—Sn1—C14	−147 (3)
C1—C2—C7—N1	88.7 (10)	C6—C1—Sn1—Cl3	−154.2 (6)
C3—C2—C7—N1	−95.6 (8)	C2—C1—Sn1—Cl3	25.0 (8)
Sn1—C14—C15—C16	154 (8)	C6—C1—Sn1—Cl1	−63.6 (6)
C14—C15—C16—C17	52 (13)	C2—C1—Sn1—Cl1	115.5 (7)
Sn1—C14B—C15B—C16B	−179 (5)	C6—C1—Sn1—Cl2	116.6 (6)
C14B—C15B—C16B—C17B	−174 (6)	C2—C1—Sn1—Cl2	−64.2 (7)
Sn1—C14C—C15C—C16C	−164 (7)	C15C—C14C—Sn1—C1	−147 (7)
C14C—C15C—C16C—C17C	−47 (12)	C15C—C14C—Sn1—C14	54 (9)
C10—C8—N1—C7	−160.6 (7)	C15C—C14C—Sn1—Cl3	31 (13)
C9—C8—N1—C7	75.3 (9)	C15C—C14C—Sn1—Cl1	−60 (11)
C10—C8—N1—C11	66.2 (9)	C15C—C14C—Sn1—Cl2	121 (11)
C9—C8—N1—C11	−57.9 (10)	C15—C14—Sn1—C14B	−91 (12)
C2—C7—N1—C8	49.8 (9)	C15—C14—Sn1—C1	−101 (6)
C2—C7—N1—C11	−176.6 (7)	C15—C14—Sn1—C14C	−72 (18)
C13—C11—N1—C8	−126.3 (9)	C15—C14—Sn1—Cl3	86 (6)
C12—C11—N1—C8	108.2 (9)	C15—C14—Sn1—Cl1	−5(6)
C13—C11—N1—C7	99.7 (9)	C15—C14—Sn1—Cl2	175 (6)
C12—C11—N1—C7	−25.8 (11)		

### Hydrogen-bond geometry (Å, °)

**Table:**

*D*—H···*A*	*D*—H	H···*A*	*D*···*A*	*D*—H···*A*
N1—H1···Cl2	0.86 (1)	2.37 (7)	3.208 (8)	165 (8)
